# Necrotizing Scleritis in Granulomatosis With Polyangiitis: A Clinical Challenge for an Ophthalmologists

**DOI:** 10.1155/crop/5056070

**Published:** 2026-07-01

**Authors:** Poonam Lavaju, Sangeeta Shah, Ashmita Jha, Binay Kamat, Kushal Gurung

**Affiliations:** ^1^ Department of Ophthalmology, B.P. Koirala Institute of Health Sciences, Dharan, Nepal, bpkihs.edu; ^2^ Consultant Ophthalmologist, Dhaulagiri Provincial Hospital, Baglung, Nepal

**Keywords:** granulomatosis with polyangiitis, necrotizing scleritis, scleritis, staphyloma

## Abstract

**Introduction:**

Granulomatosis with polyangiitis (GPA) is a granulomatous disease with multisystem involvement, frequently with ocular manifestations. It can lead to ocular morbidity due to tissue melting and necrosis.

**Case Description:**

A 35‐year‐old male presented with a painless nodular lesion in the left eye, along with gradual diminution of vision and redness for 2 months. Eight months ago, he was diagnosed with GPA and left eye anterior uveitis. He was treated with topical steroids, topical cycloplegic, pulse cyclophosphamide, oral prednisolone, and azathioprine. At presentation, best corrected visual acuity in the right eye was 6/6 and 1/60 in the left. Left eye showed scleral thinning and necrosis in the superonasal quadrant with +1 cells in the anterior chamber. Intraocular pressure was 10 mmHg. Funduscopy examination showed exudative retinal detachment with hyperemic disc. Since refractory to topical and systemic immunomodulators, he underwent corneoscleral patch graft in the left eye for the progressive scleral thinning along with injection rituximab. Significant improvement was seen at 1 month with corneoscleral patch graft in situ, decreased inflammation with formed anterior chamber depth. However, at 2 months follow‐up, the left eye showed worsening of ocular symptoms with progressive scleral thinning, necrosis, and graft lysis with total retinal detachment.

**Conclusion:**

GPA‐associated necrotizing scleritis is challenging. A collaborative timely management with aggressive compliant immunosuppressive therapy is a necessity to avoid ocular morbidity.

## 1. Introduction

Granulomatosis with polyangiitis (GPA) is a multisystemic necrotizing granulomatous vasculitis affecting small to medium‐sized vessels [1, 2]. Ocular involvement is seen in 58%–64% of cases, with varied presentations. It may be the only initial presentation, pointing towards the diagnosis [2, 3]. Ophthalmic presentations include peripheral ulcerative keratitis (PUK), episcleritis, conjunctivitis, necrotizing scleritis (NS), corneoscleral ulceration, uveitis, optic neuritis, retinal artery occlusion, orbital mass, and scleritis being the most common manifestations [1–5]. Scleritis preceded systemic disease diagnosis in 38.7% of patients with scleritis and vasculitis [6]. Necrotizing variants of GPA‐associated scleritis have been reported in 71%–86%. Scleral and corneal thinning leading to staphyloma and perforation are not unusual in GPA [7–9].

Management of NS associated with GPA remains a major challenge, sometimes requiring timely and adequate therapy. Medical as well as surgical interventions with the use of allografts and amniotic membrane grafting have been successfully reported in the literature [3, 5–8]. Multiple modalities of treatment have been suggested in the literature for varied presentations, switching between different treatment regimens and use of aggressive systemic immunomodulators. Rituximab (RTX) has been shown beneficial in the treatment of refractory cases [10–13].

Herein, we report a case of GPA (c‐ANA positive) with NS recalcitrant to conservative management requiring corneoscleral patch grafting and aggressive immunomodulatory therapy.

## 2. Case Report

A 35‐year‐old male presented with complaints of redness, a painless bluish‐black nodular lesion over the nasal part of the white part of the left eye (LE) with gradual progressive diminution of vision for 2 months.

Ten months ago, he had presented to the emergency department with cough for 1 month and fever for 1 week, followed by hemoptysis and epistaxis for 3 days, for which he was admitted to the pulmonology department. Based on contrast‐enhanced CT scan findings of multiple lung nodules, a thick‐walled cavity in the right upper lobe with pericavitatory consolidation, and patchy and nodular areas of consolidation in the right upper and left lower lobes, along with positive anti‐PR3 (14.39 U/mL), c‐ANCA, and ANA (7.54 units) tests, a diagnosis of GPA was made; this was later confirmed by positive lung and right lateral wall of the nasal cavity biopsies.

He had received intravenous 1 gm of methylprednisolone followed by six cycles of cyclophosphamide 750 mg (for organ‐threatening disease), with the first three cycles administered at 2‐week intervals and the subsequent three cycles at 4‐week intervals. Ophthalmology examination then revealed LE anterior uveitis, with visual acuity of 6/6 and 6/36 in the right (RE) and LE, respectively. He was treated with topical cycloplegics and topical steroids in tapering doses in the LE. The patient symptomatically improved and was hemodynamically stable at the time of discharge. He was discharged on tapering dose of oral prednisolone, azathioprine (75 mg, tapered to 25 mg daily), and Bactrim DS Bd for 14 days.

Unfortunately, the patient was lost to follow‐up for 6 months, after which he presented with the present ocular complaint and worsening of visual acuity in the LE. There was no history of floaters, flashes, and any ocular injury.

Respiratory system examination revealed intracapsular and axillary coarse crepitations in the right lung. Other systemic examinations and vitals were within normal limits.

**Figure 1:**
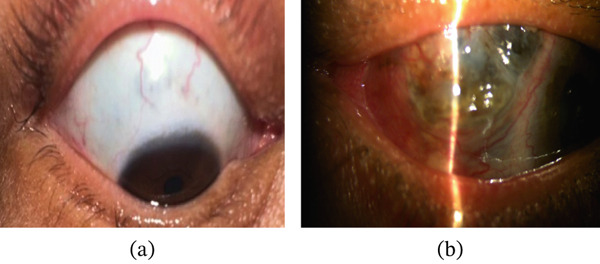
(a) Right eye and (b) left eye showing necrotizing scleritis with staphyloma (12 × 11 mm) tortious vessels.

On ocular examination, the best corrected visual acuity was 6/6 in the RE and 1/60 in the left (LE). RE findings were unremarkable (Figure 1a). Slit lamp examination of the LE showed ciliary congestion with tortuous conjunctival and episcleral vessels with bluish nodular scleral mass in the superior nasal quadrant, measuring approximately 12 × 11 mm with surrounding scleral thinning, inflammation, and necrosis (Figure 1b). Peripheral cornea showed conjunctivalization extending from 6:30 to 9 o′clock along with 360° superior vascularization and deep vascularization at 3 o′clock and 8–9 o′clock, anterior chamber reactions (cells +1, flare 1+), pupil sluggishly reacting direct and consensual reaction with posterior synechiae at 12–2 o′clock position and 5 o′clock position. Blue dot cataract was present in both the eyes. Dilated fundoscopy examination of the LE revealed superotemporal exudative retinal detachment with hyperemic disc and tortuous retinal vessels. Intraocular pressure measured 14 mm Hg in the RE and 10 mm Hg in the LE. He was diagnosed as having LE NS with exudative retinal detachment secondary to GPA.

Despite stepping up oral corticosteroids, azathioprine and topical immunomodulator (1% cyclosporine eye drops) there was progressive scleral thinning and necrosis. He underwent LE full thickness corneoscleral patch graft measuring 12 × 12 mm (sclera: 10 × 12 mm and cornea: 2 × 11 mm). The graft was secured with interrupted 10–0 ^′^ nylon sutures. Postoperative, he was prescribed with 1% prednisolone phosphate (hourly), 0.5% moxifloxacin q.i.d, 1% atropine t.i.d, 1% cyclosporine‐A eye drops b.d, and preservative free lubricating eye drops. After the rheumatologist consultation, he received two infusions of RTX 500 mg per dose two doses, 4 weeks apart along with oral steroids 1 mg/kg/day.

**Figure 2:**
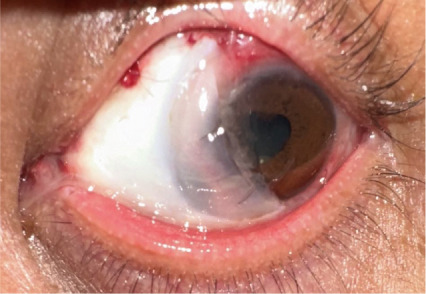
Left eye: 1‐month postoperative with corneoscleral graft in situ.

The patient significantly improved at 1‐month posttherapy with corneoscleral patch graft in situ with formed anterior chamber and decreased inflammation (Figure 2). The treatment was continued for subsequent follow‐up. After 2 months, he again presented with worsening of symptoms and decreased visual acuity in the LE.

**Figure 3:**
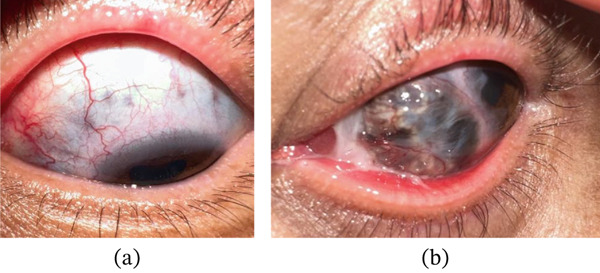
(a) Two‐month follow‐up: right eye showing patches of scleral thinning. (b) Left eye: 2 months postoperative showing scleral thinning and graft lysis.

Visual acuity was 6/9 in the RE and hand movements in the left. Examination of the RE showed patches of scleral thinning in the superior quadrant and progressive scleral thinning, necrosis with lysis of the corneoscleral graft with total exudative retinal detachment in the left (Figure 3a,b). Other findings in the RE were unremarkable. He was planned for the third cycle of RTX, but the patient refused due to financial constraints. The patient was lost to follow‐up. On query, it was found that the patient had expired after 2 months due to respiratory complications.

## 3. Discussion

GPA may present with a wide range of clinical manifestations with varying severity and complications frequently with ocular involvement. The incidence of ocular involvement in GPA varies from 29% to 79% [1–10]. NS is strongly associated with systemic disease and is seen in more than half of the patients with ocular Wegner′s granulomatosis, nearly 71%–86% [6, 9, 11, 14].

Scleritis microangiopathy in GPA is suggestive of immune complex reaction (Type III hypersensitive reaction) where vascular injury is the cause of antigen–antibody reaction that occurs inside and outside of the vessel wall, which causes activation of complement, attraction of neutrophils, and necrosis of vessels and surrounding tissue [2, 3].

NS, characterized by scleral thinning and a bluish appearance from visibility of the underlying choroid, can lead to scarring, infection, and, in advanced cases, can lead to staphyloma, perforation of the globe, and phthisis bulbi if not treated promptly or adequately [1–3]. Positive c‐ANCA in GPA has been reported in 75% of cases. Positive c‐ANCA indicates 97% specificity for GPA. However, the sensitivity of c‐ANCA varies, with 88% in patients with active generalized GPA, 50% in the active phase of limited GPA, and 32%–43% in remission [14–16]. It may aid in diagnosis of GPA; however, negative ANCA is insufficient to reject the diagnosis [14, 16].

Visual loss in patients with an established diagnosis of GPA has been documented by several authors [4–6]; however, visual loss as the primary presentation of the disease has only been reported in a small number of cases [17, 18]. Visual outcomes are variable and largely depend on pre‐existing corneoscleral involvement and associated other ocular complications [4, 9, 11]. Despite aggressive immunosuppression, visual morbidity may occur in 85% of individuals with severe necrotizing posterior scleritis if the disease is not addressed on time [1, 5, 9, 14].

The literature on corneoscleral patch graft for GPA associated NS is limited to only a few case reports, case series, and original articles. The main purpose of the surgical intervention being tectonic support and restoration of globe integrity in eyes with progressive scleral thinning and corneoscleral melt or perforation [4–6, 8, 9, 11, 19, 20].

In cases of NS, 16%–15% may land up with scleral melting requiring scleral patch graft, with varied prognosis and lysis of the graft [9, 11].

Early recognition and subsequent aggressive medical therapy and surgical reinforcement using allografts, not only to control inflammation of the eye but also to prevent potentially life‐threatening complications and ocular morbidity [2, 5, 9]. Successful management of scleral thinning has been reported in the literature with the use of allografts and amniotic membrane grafting [2, 4, 5, 9, 11].

Corneoscleral patch grafting offers good anatomical prognosis in GPA associated NS, with reported success rates of approximately 77%–80% [4–6, 9, 11, 19, 20]. Sangwan et al. reported tectonic success in 76.9% (10/13 eyes) undergoing scleral patch grafting for scleral defects including NS, with preservation of globe in most of the eyes over a mean follow‐up of approximately 2 years [20]. Reportedly, Tenon′s patch graft, oral mucosal graft, pericardial grafts, dermal grafts, fascia‐lata, periosteum, and synthetic materials like Gore‐tex had been reported as an alternative to corneoscleral patch graft [19, 20].

In a study by Mohanan‐Earatt et al., 17 patients (19 eyes) with GPA associated with scleritis were included, out of which 15 eyes had NS. Four eyes with NS associated with peripheral keratitis, of which three required scleral patch graft in addition to immunosuppressive therapy. However, one patient subsequently developed graft lysis, which was managed well with amniotic membrane grafting and immunomodulators [11].

Similarly, Gu J et al. studied seven eyes of GPA associated with NS. Five patients required surgical intervention, of which two cases of NS (three eyes) received autologous sclera grafts, two cases of PUK were treated with lamellar keratoplasty, and one case of paracentral corneal perforation was repaired with a tectonic intralamellar patch plus an on‐lay graft along with oral cyclophosphamide. A total of 78% of the patients with NS and/or PUK with progressive inflammation was halted by the combination of immunosuppressive and surgical therapy. Despite treatment with systemic corticosteroids and methotrexate, scleral graft lysis was evident in one of the eyes within 1 month, which was managed with the addition of oral cyclophosphamide and amniotic membrane transplantation. AMT acts only as an adjunct to scleral grafting, as it promotes re‐epithelization and vascularization. One of the patients died of respiratory failure [9].

Similarly, Sainz de la Maza et al. have demonstrated stable graft survival when combined with systemic immunosuppressive therapy, whereas graft lysis occurred with withdrawal of the therapy [6].

Hence, literature supports the role of corneoscleral patch grafting as an effective globe preserving procedure in cases with severe scleral thinning or perforation, while emphasizing the need for adequate control of the underlying inflammation with corticosteroids and immunomodulators for the long‐term prognosis of corneoscleral grafts, to prevent recurrence and graft necrosis [4, 6, 10, 11, 13, 19, 20].

Multiple modalities of treatment have been suggested in the literature, switching between different treatment regimens and with use of aggressive systemic immunomodulators, switching to RTX from cyclophosphamide or vice versa [1, 4, 9, 11, 13, 21].

Different studies have demonstrated the efficacy of RTX over cyclophosphamide in treating GPA with ocular involvement, particularly in refractory NS and in those with orbital granulomas [3, 4, 10, 13]. However, some of the previous results still fail to do so, requiring additional doses of immunosuppressive therapy [3, 9, 11–13].

In a larger single‐center study of 63 patients, Pérez‐Jacoiste Asín et al. were able to show successful outcome with RTX over cyclophosphamide especially in refractory cases. Ocular remission occurred in 11/12 eyes (91.7%) recalcitrant to other medications [4]. Joshi and Mittal reported an 80% remission rate in patients with ocular GPA who received RTX. Localized disease and prior cyclophosphamide treatment may influence the risk of relapse [7].

Recillas‐Gispert et al. have shown that RTX is useful in the treatment of NS associated with GPA in patients who go into relapse after remission, with remission achieved in 86%. Two patients required a second dose of RTX to treat ocular relapse, with promising results. One patient with posterior involvement developed phthisis bulbi and another had ocular perforation needing enucleation to prevent endophthalmitis [13].

Fujita et al. reported successful remission with use of intravenous RTX in adjunction with AMT in a 67‐year‐old having PUK associated with GPA, recalcitrant to intravenous injection of methylprednisolone, and intravenous injection of cyclophosphamide [21].

In our case, progressive scleral thinning and necrosis were noted even with the use of conservative modalities requiring corneoscleral patch grafting in addition to the use of RTX. The patient was doing well until 1‐month follow‐up. However, after 1 month, the patient developed graft lysis with total retinal detachment in the LE and patches of scleral thinning in the RE. Due to financial constraints, he refused to go ahead with further necessary treatment. Later on, inquiry, it was found that the patient had expired due to respiratory failure. Therapeutic withdrawal and poor compliance may be the cause of the poor prognosis and mortality.

## 4. Conclusion

GPA‐associated NS can be very challenging; necessitating early diagnosis to eliminate potential complications. A collaborative management with rheumatologist is a necessity, keeping in mind the necessity of prolonged therapy and financial constraints.

## Author Contributions

Poonam Lavaju did the substantial diagnosis and management of the case. Sangeeta Shah gave the additional support and guidance in the management of the case. Kushal Gurung, Ashmita Jha and Binay Kamat made contributions in daily workup and monitoring of the patient.

## Funding

No funding was received for this manuscript.

## Ethics Statement

Ethical approval has been obtained from Intuitional Review Committee, BPKIHS, Dharan, Nepal to proceed for presentation and publication.

## Consent

All the appropriate consent has been taken from the patient for the images and other clinical information to be reported in the journal. The patient knows that the identity will not be disclosed.

## Conflicts of Interest

The authors declare no conflicts of interest.

## Data Availability

The data that support the findings of this study are available on request from the corresponding author. The data are not publicly available due to privacy or ethical restrictions.
